# Effect of steam explosion of oil palm frond and empty fruit bunch on nutrient composition and ruminal fermentation characteristics

**DOI:** 10.1007/s11250-019-02117-4

**Published:** 2019-11-09

**Authors:** Hao Wu, Zhenming Zhou, Yuanlong Yang, Qingxiang Meng

**Affiliations:** State Key Laboratory of Animal Nutrition, College of Animal Science and Technology, China Agricultural University, Beijing, 100193 China

**Keywords:** Steam explosion, Oil palm by-products, Nutrient composition, Gas production, Ruminal fermentation in vitro

## Abstract

In this study, we evaluated the effect of steam explosion of oil palm frond (OPF) and oil palm empty fruit bunch (EFB) on nutrient composition and ruminal fermentation characteristics in vitro. The results showed that steam explosion decreased NDF (*P* < 0.01), ADF (*P* < 0.01), and hemicellulose content (*P* < 0.01) in OPF and EFB. Steam explosion improved the effective energy value of OPF and EFB. In vitro fermentation results revealed that 72-h gas production capacity of OPF and EFB increased by 12.60and 85.06% (*P* < 0.01), respectively, after steam explosion. Steam explosion had a tendency to improve the concentration of total volatile fatty acids (TVFA) (*P* = 0.082). In conclusion, steam explosion of OPF and EFB reduced NDF, ADF, and hemicellulose content and increased gas production and TVFA concentration.

## Introduction

Oil palm (*Elaeis guineensis*), which is native to Western Africa, grows in tropical regions and is considered to be the most important agricultural crop in China and Southeast Asia. As palm oil production increases, oil palm by-products such as empty fruit bunch (EFB), palm kernel shell (PKS), oil palm trunk (OPT), and oil palm frond (OPF) grow exponentially. Oil palm constitutes a renewable cellulose source; however, due to local heat and highly perishable by-products, the disposal of oil palm residues represents a significant concern for the oil palm industry. Currently, by-products of oil palm are incinerated or used as organic fertilizer, thereby leading to increased environmental pollution and costs (Abnisa et al. 2013). Therefore, it is important to find applications for oil palm by-products to improve their economic value. Steam explosion is commonly used in the biomass resources because most of the hemicellulose can be extracted from the exploded materials (Martin-Sampedro et al. 2012). Moreover, steam explosion enhances the accessibility of bacteria and enzymes to the exploded materials. Steam explosion of oil palm by-products may potentially improve their digestibility. Most studies on steam explosion of biomass resources have focused on industrial applications such as pulping, saccharification, and fermentation. There is limited information on steam-exploded products and their digestion in ruminant animals. Therefore, this study assessed the effects of steam-exploded oil palm by-products on chemical nutrient composition and ruminal fermentation characteristics.

## Materials and methods

### Animals and diets

All animal experiments were approved by the Animal Welfare and Ethical Committee of China Agricultural University (Permit No. DK18030608) and performed in accordance with the Regulations for the Administration of Affairs Concerning Experimental Animals (The State Science and Technology Commission of P. R. China, 1988). Ruminal fluid was collected and pooled from three Simmental × Fuzhou crossbred steers (average body weight is 400 kg), each fitted with a permanent rumen cannula. The ingredients and nutrient composition of the experimental diets are shown in Table 1. Ruminal fluid inoculum was obtained prior to the morning feeding. Ruminal contents were passed through four layers of cheesecloth, pooled, and sent to the laboratory.

**Table 1:** Ingredients and chemical composition of the diet

Items	%, DM
Ingredients	
Steam-flaked corn	34.10
Soybean curb residue	12.60
Brewer’s grains	8.10
Maize silage	39.00
Chinese wild rye	5.00
Sodium bicarbonate	0.30
Salt	0.50
Premix^1^	0.50
Chemical composition	
ME(MJ/kg)	6.35
OM	93.91
CP	13.02
ADF	29.27
NDF	53.86
Ca	0.61
P	0.29

*ME* metabolic energy, *OM* organic matter, *CP* crude protein, *ADF* acid detergent fiber, *NDF* neutral detergent fiber

^1^Premix contained the following (IU per kg): vitamin A, 3000; vitamin D_3_, 1200; vitamin E, 10; (mg): Cu, 8; Fe, 50; Zn, 30; Mn, 40; Co, 0.1; Se, 0.2; I, 0.5

### Chemical analysis and FE-SEM analysis

We obtained OPF and EFB samples from Hainan, China, and randomly divided them into two parts. One part was sun-dried at ambient temperature for a several days. The samples were subsequently ground to a 10~20-mm particle size using a knife mill, and rubbed. The other part was subjected to steam explosion after soaking in water for 24 h. Steam pressure was 1.5 MPa and the retention time was 1 min. The treatment was conducted by using a specially designed steam explosion vessel at the pilot plant of the Beef Cattle Experimental Station of China Agricultural University. Steam explosion was conducted three times. Each batch of about 3 kg of OPF or EFB was put into the steam chamber. The steam was adjusted to the desired pressure 1.5 MPa. Counting of retention time 1 min for each run was started when steam reached to the target pressure. Steam was suddenly released at the end of each treatment to give the explosion effect. The treated OPF or EFB was collected, dried, and ground as described above.

The chemical composition of OPF and EFB (Table 2) was determined following AOAC methods (2012). Crude protein (CP) was determined by measuring nitrogen content (FP-528, Leco, St. Joseph, MO, USA). Crude fiber (CF), neutral detergent fiber (NDF), acid detergent fiber (ADF), and lignin (ADL) were measured using a fiber analyzer (A220, Ankom Technology, Macedon, NY, USA). Ether extract (EE) was analyzed by an extraction system (Ankom XT10 extractor, Ankom Technology, Macedon, NY, USA). Calcium was determined by atomic absorption spectrophotometry (WFX-320, Braic, Beijing, China), and phosphorus was determined by UV spectrophotometry (UVVIS 8500, Tianmei Scientific Instrument Co., Ltd., Shanghai, China). Based on the nutrient content of the feeds, we used the energy value prediction formula of INRA (1978, 1988) to calculate the effective energy. The formulas included GE (Mcal/kg) = 17.3 + 0.0617 × CP + 0.2193 × EE + 0.0387 × CF − 0.1867 × ash + 0.19; DE (Mcal/kg) = GE × Ed/100; ME (Mcal/kg) = DE × ME/DE; NE_m_ (Mcal/kg) = ME × km; and NE_l_ (Mcal/kg) = ME × kl; NE_g_ (Mcal/kg) = ME × kg, where GE is gross energy, DE is digestible energy, ME is metabolizable energy, NE_m_ is the net energy for maintenance, NE_l_ is the net energy for lactation, and NE_g_ is the net energy for weight gain.

**Table 2:** Effect of steam explosion on nutrient composition of oil palm frond (OPF) and oil palm empty fruit bunch (EFB) (%, DM)

Item	OPF		EFB		SEM	P		
	Control	Steam explosion	Control	Steam explosion		S	T	S × T
DM	97.57^b^	97.69^a^	92.55^d^	93.32^c^	0.024	< 0.001	< 0.001	< 0.001
CP	1.98^b^	1.95^b^	4.54^a^	4.72^a^	0.098	< 0.001	0.455	0.321
EE	2.77^c^	1.33^d^	4.62^b^	8.22^a^	0.119	< 0.001	< 0.001	< 0.001
NDF	73.26^b^	65.69^c^	80.86^a^	72.12^b^	0.424	< 0.001	< 0.001	0.208
ADF	49.22^c^	47.46^d^	56.00^a^	53.89^b^	0.434	< 0.001	0.002	0.692
ADL	9.72^c^	9.45^c^	16.21^a^	15.88^b^	0.100	< 0.001	0.016	0.768
Ash	3.31^d^	4.19^c^	5.40^a^	5.12^b^	0.018	< 0.001	< 0.001	< 0.001
Hemicellulose	22.81^b^	18.81^c^	24.39^a^	18.76^c^	0.435	0.116	< 0.001	0.100
Cellulose	38.61^a^	34.38^b^	38.35^a^	35.99^b^	0.511	0.223	< 0.001	0.103

S represents the oil-palm by product, T represents steam explosion, and S × T represents the interaction effect between S and T. In the same row, values with different letters represent significant differences (*P* < 0.05)

*DM* dry matter, *CP* crude protein, *EE* ether extract, *NDF* neutral detergent fiber, *ADF* acid detergent fiber, *ADL* acid detergent lignin

SEM analysis was performed with a MERLIN FE-SEM (Carl Zeiss Microscopy, Oberkochen, Germany). The samples were AU-coated by sputtering method using a JEOL JFC-1600 coater sputter (Redding, USA).

### In vitro gas production and fermentation parameters

Incubations were conducted anaerobically using the gas production method reported by Menke et al. (1979). Gas production was repeated three times for each group. Briefly, ruminal fluid filtrates were pooled into an anaerobic buffer solution under a constant flow of O_2_ that was free of CO_2_ (ruminal fluid-to-buffer ratio 1:2). Using an automatic pump, the inoculated culture medium were transferred into glass syringes (HFT000025, Häberle Maschinenfabrik GmbH, Germany), which were previously warmed to 39 °C. The syringes were incubated in a shaking water bath at 39 °C. Blank syringes containing only inoculated culture medium with no substrate were simultaneously incubated. There were three blank syringes per group. Gas production in each syringe was measured at 1, 2, 3, 4, 5, 6, 8, 10, 12, 16, 20, 24, 28, 32, 36, 40, 48, and 72 h. To terminate the fermentation, the syringes were placed in ice water. To assess fermentation, duplicate syringes were incubated as described above; however, the incubation was terminated at 24 h. Aliquots of the fermentation fluid were sampled for pH measurement (10 mL) and for VFA and ammonia N determination (10 mL) prior to centrifugation at 8000*g* for 15 min at 4 °C. The dynamics of gas production were computed by the nonlinear equation (Orskov and Mcdonald 1979) *y* = *b* × (1 − *e*^−*ct*^), where *y* is the volume of gas produced at time *t*, *b* is the potential gas production (mL/g DM), and *c* is the fractional rate of gas production.

### Statistical analysis

We analyzed the data using SAS (version 9.4; SAS Institute, Cary, NC, USA) software. Nutrient composition, gas production, and in vitro fermentation characteristics were analyzed using the MIXED procedure with the following model *y*_*ij*_ = *μ* + *α*_*i*_ + *β*_*j*_ + *α*_*i*_ × *β*_*j*_ + *e*_*ij*_, where *y*_*ij*_ is the dependent variable, *μ* is the overall mean, *α*_*i*_ is the fixed effect of steam explosion, *β*_*j*_ is the fixed effect of oil palm by-product, *α*_*i*_ × *β*_*j*_ is the interaction between treatment and oil palm by-product, and *e*_*ij*_ is the random residual error. Duncan significant difference test was performed to determine differences among means. Statistical significance was set at *P* ≤ 0.05.

## Results

### Effect of steam explosion on the nutritional composition of OPF and EFP

The nutrient composition of OPF and EFP is shown in Table 2. Steam explosion reduced NDF and ADF content (*P* < 0.01); however, the interaction between steam explosion and oil palm by-product type was not significant (*P* > 0.05). In OPF and EFB, NDF content decreased from 73.26 and 80.86% to 65.69 and 72.12%, respectively, and ADF content decreased from 49.22 and 56.00% to 47.46 and 53.89%, respectively. Steam explosion also reduces cellulose and hemicellulose content in OPF and EFB (*P* < 0.01). Following steam explosion, hemicellulose content decreased from 22.81 to 18.80% in OPF and from 24.39 to 18.80% in EFP. Cellulose content was reduced from 38.61 to 34.38% in OPF and from 38.35 to 35.99% in EFB. Additionally, steam explosion increased EE content in EFB (*P* < 0.01) and reduced EE content in OPF (*P* < 0.01). Steam explosion and oil palm by-products had significant interaction effects (*P* < 0.01). There were no significant differences in CP between control and treatment (steam explosion) and no significant interaction effects among palm by-products types (*P* > 0.05).

### Effect of steam explosion on the effective energy value of OPF and EFB

The prediction of energy value is an important aspect of the evaluation of the nutritional value of ruminant feed. Table 3 shows the changes in GE, DE, ME, NE_m_, NE_l_, and NE_g_ following steam explosion of OPF and EFB. Steam explosion numerically increased the effective energy value of OPF and EFB. Following steam explosion, ME, NE_m_, NE_l_, and NEg increased by 1.26, 1.90, 1.99, and 5.26%, respectively, in OPF and by 13.52, 14.99, 14.99, and 24.06, respectively, in EFB.

**Table 3:** Effect of steam explosion on energy values of oil palm frond (OPF) and oil palm empty fruit bunch (EFB)

Item	OPF		EFB	
	Control	Steam explosion	Control	Steam explosion
GE (MJ/kg)	19.63	19.09	20.03	20.81
DE (MJ/kg)	8.90	9.00	7.27	8.24
ME (MJ/kg)	7.17	7.26	5.77	6.55
NE_m_ (MJ/kg)	4.73	4.82	3.67	4.22
NE_l_ (MJ/kg)	3.95	4.03	3.07	3.52
NE_g_ (MJ/kg)	2.09	2.20	1.33	1.65

*GE* gross energy, *DE* digestible energy, *ME* metabolic energy, *NE*_*m*_ net energy for maintenance, *NE*_*l*_ net energy for lactation, *NE*_*g*_ net energy for weight gain

### Effect of steam explosion on the morphologies of OPF and EFB

**Fig. 1:**
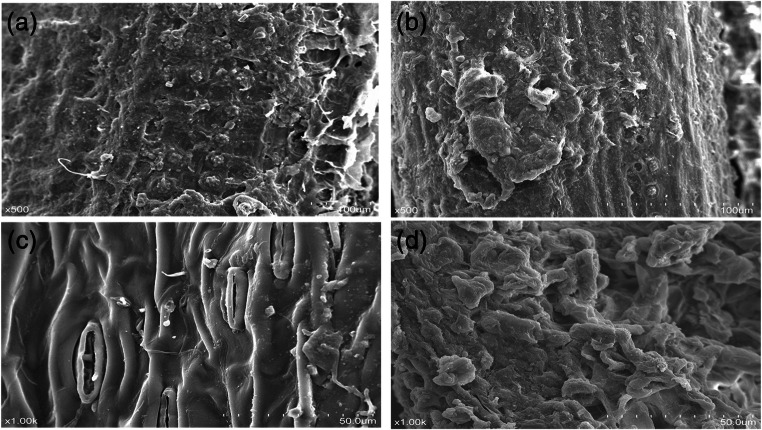
The surface morphology of all the samples performed by SEM. In untreated empty fruit bunch (EFB), the straw itself is surrounded by a sheath (**a**), and SEM scan of EFB surface shows the “globular” deposits characteristic (**b**). In untreated oil, palm frond (OPF) (**c**) and steam explosion causes partially separated fiber and a surface layer with debris and droplets (**d**)

Changes of morphologies of the steam exploded materials (A and C) in comparison with the original materials (B and D) are presented in Fig. 1. In general, the regular shape of the sample changed into spherical and round shaped particles after steam explosion. Moreover, there were more free granules sticking to the surface.

### Effect of steam explosion on gas production and fermentation in vitro

Table 4 shows the effect of steam explosion of OPF and EFB on in vitro gas production. The 72-h gas production capacity of OPF and EFB increased by 12.60% and 85.06% (*P* < 0.01), respectively, after steam explosion. There was a significant interaction between steam explosion and oil palm by-products (*P* < 0.01). The predicted maximum gas production volume increased from 99.50 to 107.75 mL in OPF (+ 8.29%) and from 33.24 to 62.06 mL in EFB (+ 86.70%, *P* < 0.01). There was no significant effect on gas production rate (*P* > 0.05) among oil palm by-products after steam explosion.

**Table 4:** Effect of steam explosion on the 72-h gas production (GP) of oil palm frond (OPF) and oil palm empty fruit bunch (EFB)

Items	OPF		EFB		SEM	P		
	Control	Steam explosion	Control	Steam explosion		S	T	S × T
72 h GP (mL)	105.13b	118.38a	35.55d	65.79c	2.002	< 0.001	< 0.001	0.005
Predicted maximum GP (mL)	99.50b	107.75a	33.24d	62.06c	1.651	< 0.001	< 0.001	< 0.001
Rate of GP (h^−1^)	0.14a	0.14a	0.10b	0.09b	0.007	< 0.001	0.274	0.147

S represents the oil-palm by product, T represents steam explosion, and S × T represents the interaction effect between S and T. In the same row, values with different letters represent significant differences (*P* < 0.05)

Table 5 shows the effect of steam explosion on the fermentation parameters of OPF and EFB following a 24-h in vitro digestion. It was shown that steam explosion had no significant effects both on NH_3_-N concentration and pH value (*P* > 0.05). Though steam explosion had a tendency to increase the concentration of total volatile fatty acids (TVFA) (*P* = 0.082) and the molar proportion of valeric acid (*P* = 0.074), there was no treatment effect on other individual VFA (*P* > 0.05) in OPF and EFB. However, there was interaction between by-product sources and treatments on individual VFAs. Specifically, molar proportion of propionate acid was increased by steam explosion, while isobutyrate acid and isovaleric acid were decreased significantly only in EFB.

**Table 5:** Effect of steam explosion on fermentation of oil palm frond (OPF) and oil palm empty fruit bunch (EFB)

Items	OPF		EFB		SEM	P		
	Control	Steam explosion	Control	Steam explosion		S	T	S × T
pH	7.24	7.16	7.18	7.12	0.049	0.341	0.176	0.845
NH_3_-N (mg/100 mL)	20.36b	22.31b	27.66a	25.34a	0.852	< 0.001	0.829	0.037
TVFA (mmol/L)	30.61b	35.96a	28.72b	29.35b	1.503	0.022	0.082	0.155
VFA molar proportion (%)								
Acetate	67.12a	67.27a	64.35b	65.71ab	0.687	0.014	0.307	0.408
Propionate	21.11ab	20.66ab	20.52b	21.59a	0.322	0.616	0.358	0.046
Isobutyrate	7.35ab	7.58ab	7.66a	7.26b	0.113	0.966	0.471	0.022
Butyrate	1.64b	1.63b	3.51a	2.29b	0.370	0.009	0.135	0.143
Isovalerate	0.72a	0.78a	0.80a	0.68b	0.035	0.767	0.423	0.031
Valerate	2.07b	2.08b	3.16a	2.48b	0.163	0.002	0.074	0.064
A:P	3.18	3.26	3.14	3.05	0.072	0.119	0.924	0.282

S represents the oil-palm by product, T represents steam explosion, and S × T represents the interaction effect between S and T. In the same row, values with different letters represent significant differences (*P* < 0.05)

*TVFA* total volatile fatty acids, *A:P* molar ratio between acetate and propionate

## Discussion

OPF and EFB consist mainly of cellulose, hemicellulose, and lignin. Steam explosion may dissolve most hemicellulose in raw materials and degrade a small amount of cellulose and lignin, which will contribute to improved enzymatic accessibility (Silveira et al. 2018). The collapse surface morphology was observed for OPF and EFB, which was probably due to the substantial depolymerization and decreased solubilization of lignin in steam explosion. During steam explosion, hemicellulose is hydrolyzed into monosaccharides and oligosaccharides, the crystallinity and polymerization of cellulose are reduced, and lignin is degraded into phenolic acids (Carvalho et al. 2018; Tanpichai et al. 2019). Additionally, under high temperature conditions, acetyl cellulose yields acetic acid, which accelerates the degradation of cellulose and hemicellulose (Carvalheiro et al. 2008). These factors eventually decrease the content of cellulose and hemicellulose in oil palm by-products. Our results were consistent with those obtained by others (Chang et al. 2012), who reported that cellulose, hemicellulose, and lignin were reduced by 8.47%, 50.45%, and 36.65%, respectively, in steam-exploded corn straw. As a result of reducing crude fiber but crude protein content of the materials, crude fat was more likely concentrated other than increased by steam explosion in EFB. Chaji et al. (2010), who used in vitro gas production technique to predict the energy value of bagasse, reported that ME increased by 44.43% after steam explosion treatment, which was the same with our findings. The effective energy values of the samples were predicted base on the chemical compositions using the equations of INRA, where the EE content made a major contribution to the results. Take EFB as example, steam explosion increased the EE, and decreased the CF content of the material, which could make the calculated GE value higher than untreated sample.

Ruminal bacteria perform a number of metabolic functions that are essential for animal physiology and performance, including nutrient metabolism, polysaccharide degradation, and fermentation (Welkie et al. 2010; Derakhshani et al. 2017). Steam explosion significantly improved gas production from OPF and EFB, consistent with previous reports (Liu et al. 1999; Chaji et al. 2010). Chaji et al. (2010) reported that the 72-h maximum gas production of bagasse increased from 107.6 to 118.5 mL (+ 10.13%) following steam explosion. Similarly, Liu et al. (1999) observed that steam explosion increased total gas production of wheat straw by 27%. There is a linear and positive relationship between soluble carbohydrate content and feed gas production (Liu and Orskov 2000). Castro et al. (1994) concluded that in vitro wheat straw gas production was significantly higher in the steam-exploded group than in the control group (49.8 mL vs. 33.2 mL) (Castro et al. 1994). The increase in gas production may be attributed to an increase in soluble carbohydrate content in the feed. Steam explosion could increase soluble oligosaccharides, which served as an energy source for microorganisms, represented a high degradation of hemicellulose in the feed (Sabiha-Hanim et al. 2015). Moreover, steam explosion may denature the cuticle structure of the plant, thereby reducing the crystallinity and polymerization of cellulose and improving the accessibility of cellulase to the feed. Kim et al. (2005) reported that the molecular percentages of propionate increased in steam-exploded rice straw compared to untreated rice straw, which is similar to EFB treatment (Kim et al. 2005). In general, increased propionate in the rumen accompanies increase in feed efficiency because propionate is gluconeogenic in animal tissues and much of it is converted to energy for animal growth. The increase in gas production indicated that nutrients in the feed are better utilized by microorganisms; therefore, steam explosion may improve the nutritional value of OPF and EFB.

Ruminal bacterial adherence plays important roles in feed digestion. Minato et al. (1993) reported that microbial populations associated with feed particles are estimated to be responsible for 91% of glucanase, 88% of xylanase, 70% of amylase, and 75% of protease activity (Minato et al. 1993). The surface morphology of all the samples performed by SEM indicate that steam explosion may damage cellulose, degrade hemicellulose, and reduce crystalline and amorphous cellulose, thereby favoring the adhesion of bacteria to the feed (Akin 1989; Benghedalia et al. 1993). Steam explosion could potentially promote the digestion and absorption of feedstuff in the rumen, thereby improving its nutritional value. At the same time, the cuticle structure of the feed particles is destroyed, and the contact areas between ruminal bacteria and feedstuff increase (Orpin 1984; Miron et al. 2001), thereby improving the accessibility to feedstuff.

In conclusion, steam explosion significantly affected the nutrient content of OPF and EFB. After steam explosion, NDF and ADF significantly decreased in OPF and EFB. Steam explosion significantly improved the predicted effective energy of OPF and EFB and increased gas production in vitro and VFA concentration.
